# Book Reviews

**Published:** 1809-05

**Authors:** 


### CRITICAL ANALYSIS
### OF THE
### RECENT PUBLICATIONS
### ON THE
### DIFFERENT BRANCHES OF PHYSIC, SURGERY, AND MEDICAL PHILOSOPHY.

### *The Edinburgh Medical and Surgical Journal, No. XVIII*

Article 1.—*Remarkable Recovery from a very extensive Wound in the Abdomen. By* B. Hague, Surgeon, Ripon.

A Lad, about 12 years old, was wounded in the abdomen by a pair of wool-shearers. The incision was from the *scrobiculus cordis*, nearly in the direction of the *linea alba*, about four inches in length. From this wound, the great arch of the stomach, and the the whole of the intestinal canal, excepting the duodenum, protruded. The viscera were reduced in their proper order, beginning with the stomach. The sides of the wound were brought together by five sutures, and adhesive plaster applied in straps. The patient was bled freely and purged. By the twenty-fifth day, he was so well, that his surgeon took leave of him. The following remarks of the author, we shall give in his own words.

"*Remarks.*

"It may be matter of surprise and doubt to some, that the *stomach*, as well as the intestines, should have protruded through a wound in the abdomen, situated as described in the case just related; and it may not improbably be conjectured, that some portion of gut had really been mistaken for that viscus.

"The *great arch* with its *appending omentum*, were, however, too plain and evident to admit of any error of this sort; and my partner, Mr. A. Robinson, on seeing its form externally on the evening of the 31st, distended so low, had no doubt of the fact. Dr. Caley and Mr. Lucas both saw the lad at Ripon on the 20th, and both saw the stomach distended much below its proper situation: its protrusion, however, is readily accounted for by the manner in which the boy was conveyed home after the accident.

"Instead of being laid on a hurdle, and placed upon his back, he was taken before a man on horse back; at this time but a small portion of gut had protruded, and a handkerchief was given him, with which he was desired to press upon the wounded part, which he, quite overcome with fright and sickness, was unable to do. The horse had proceeded but a little way, before he felt more intestine pushing out, till at length such a quantity had protruded, as by its weight was sufficient to drag out the remainder, and in this way the great arch of the stomach was drawn down. He was carried in that manner two or three hundred yards.

"Nov. 20th. [About three months after the cure was compleated.] Mr. Lucas saw the boy with me to day. The stomach still remains much below its natural situation, so much so, as, with the bulging out of the intestines around the wounded part, to give that side of the abomen a very irregular, prominent appearance.

"He has been very well in every respect ever since his recovery; his appetite and digestion are both as good as formerly; and he is capable of enduring labour as well, and to as great a degree as before the accident."

Article 2.—*Observations on Lithotomy, with the account of a Case in which the operation was performed with the Knife. By* William Lawrence, Esq. Demonstrator of Anatomy at St. Bartholomew's Hospital.

Mr. Thompson, in his Observations on Lithotomy, has described, with much accuracy, the history of that operation. In shewing shewing the advantage of the gorget, as used by Cheselden, over the cutting gorget of Hawkins: Mr. Thompson contented himself with expressing his surprise, that the English surgeons should ever have adopted the latter. Mr. Lawrence seems to wonder at this surprise in Mr. Thompson; but, probably, Mr. Thompson's friends will not be at a loss to explain that gentleman's mode of expressing himself. It is most likely that he was as well aware as his commentator, or even as Mr. J. Bell. But men have different modes of saying the same thing. There may, indeed, be certain events, which, at particular times, excite stronger language; and under the impression of some such event, we conceive Mr. Lawrence wrote the paragraph before us.

After these remarks, we feel it our duty to add, that we perfectly agree with Messrs. Lawrence and Bell in their opinion, but that we are much better pleased with the manner in which Mr. Thompson has expressed himself.

Mr. Lawrence performs the operation without a gorget of any kind, and there cannot be a doubt of his ability as a practical anatomist, "to travel, as Mr. Hunter used to express himself, all through the human body with his knife and probe." It must also be admitted, that no man should undertake such an operation as lithotomy, without a complete anatomical knowledge of the parts. But there is something in habit; very able operators used the cutting gorget, we believe all the celebrated men of that celebrated school, at which Mr. Lawrence is so valuable a demonstrator. The pupils of those days learned the same, and some of them are now so far advanced in years, that it may be difficult for them to change their habits. We must also add, that even a good anatomist may find some relief from the use of the gorget, whilst introducing the forceps. And highly necessary as anatomical knowledge may be, it can hardly be expected, that a person residing at such a distance from the metropolis, as would render it necessary to perform the operation on the spot, can enjoy all those advantages, and retain all his school learning, with that accuracy which the constant habit of teaching must induce.

We transcribe the following case for the same reasons as the author relates it, namely, because the use of the knife was found adequate to every purpose under very unfavourable circumstances; and because the unexpected issue renders the history very interesting, we shall add, that the detail of it does credit to the author's candour.

"Mr. R. Cooper, aged 63, a healthy, robust, and fat man, came to me from the country to undergo the operation of lithotomy, which I performed on the first of December 1808, assisted by my friend Mr. Crowther, surgeon of Bethlem and Bridewell hospitals. The ordinary staff and common scalpel were employed, and the various steps of the operation were executed in the manner already described. No attempt was made to feel the staff in the perinæum; the great depth of fat would have rendered that effect unavailing; tailing; for my forefinger was buried beyond the middle joint, before I had exposed the groove of the staff. In prolonging the incision towards the bladder, an inconvenience was experienced from the great thickness of fat, in consequence of which nearly the whole length of the knife was buried in the wound. I would certainly be provided, in a similar case, with a scalpel of greater length. When the bladder was opened, the surface of the stone could be just felt with the finger, thrust up as high as possible into the wound. The forceps were introduced by the direction of the finger, and extracted, without the smallest resistance or difficulty, a stone, whose greatest circumference measured five inches. The blood lost during the operation was entirely venous, and not in large quantity; but a considerable hæmorrhage took place after the patient was laid in bed: this ceased on exposing the parts, and applying cold water externally. Nothing could be more favourable than the progress of the case; there was no tension nor pain about the bladder, nor any other disturbance, except the slight febrile irritation which such a wound must necessarily cause. On the morning of the 10th a copious bleeding took place from the wound, and produced a temporary debility, which had gone off on the following day. The patient now seemed perfectly well, excepting that there was still a wound in the perinæum: but on the 14th day, while he was taking the last mouthful of a hearty dinner, he suddenly died. His daughter came to see him from the country, and entered the room abruptly, when he fell back in the bed, turned extremely pale, and in about a quarter of an hour expired, without a groan or struggle. No entreaties could prevail with the relations to allow an examination of the body."

ARTICLE 3.—*Case of Erythema not occasioned by Mercury. Read before the Medical Society of Liverpool. By* JOHN RUTTER, M. D.

THIS patient had, in a mild degree, all those symptoms which have lately given rise to the terms *lepra mercurialis*, *erythema mercurialis*, and lastly, *hydrargyria*. Yet there is no proof that he had taken mercury. We must, on this occasion, remark, that the habit of giving mercury is become so general, we had almost said so indiscriminate, that it is no longer easy for any man to determine when he takes calomel. A dose of physic was formerly confined to a few well known remedies, the operation of which produced the desired effect, or none at all. At present a preparative pill must be given if a patient wishes for a purge, or perhaps he must be allowed nothing but a larger dose of this panacæa, which, whether it produces a purgative effect or not, may induce symptoms the cause of which we shall be at a loss to discover. Nothing here said is intended to undervalue Dr. Rutter's communication, which contains considerable merit. The inquiry, whether the disease is not improperly named, if it can arise without any mercurial irritation, is highly reasonable. But it should be remembered, that that Dr. Rutter's patient had been, on a former occasion, attacked with the same disease, whilst under the influence of mercury: and when an action has once been excited, it may, in many instances, be afterwards renewed by a slight degree of the cause which first induced it; as is remarkable of the sea sickness. Nor is it any objection to this opinion, that some patients were cured of this erythema, whilst they continued to use mercury. The same happens in the complaint we have used as an illustration. These hints are only offered to assist the inquiry so laudably proposed by Dr. Rutter.

Article 4.—*History of a Case of Tetanus, cured by Purgatives. By* H. Briggs, M. D. one of the Physicians of the Dispensary, Liverpool.

In this history, it is not a little remarkable, that both the nosological and therapeutic parts should be somewhat doubtful. That the patient had a violent and pretty general spasmodic affection, and that he took plenty of calomel and other purges is very certain. But the jaw never was completely locked; and in one of those constitutions which we usually call nervous, so many strange symptoms occur, that it is not easy to do more than refer them to one general name. However, the case is curious in showing how far even violent remedies may be carried without injury to a constitution, under the influence of any other impression. To do justice to the author, we shall conclude the paper with a few of his own remarks, before we offer any more of our own.

"Here, however, I should mention one symptom, which is very generally described as occurring in *either* species of tetanus, and in fact constituting one of its most distressing and dangerous concomitants, the *pain under the ensiform cartilage;* but which has not been particularly noticed in the present instance. 'Tis true, *all* the muscles of the back and belly were the parts the most severely affected, from first to last; but certainly the patient never did complain of *more pain at the pit of the stomach* than in any other part of the abdomen. It was almost constantly referred to the *lower* insertions of the muscles, where, indeed, it was most distressing; and the muscles were drawn into such knots, that my assistant, on first examining the patient, as he sat with his thighs and trunk drawn towards each other, mistook one of them for a bubo.

"But is this pain under the ensiform cartilage essential to the definition of genuine tetanus? Or rather, is it not observed more particularly when the disease has *existed for some time,* and when considerable quantities of *opium* had been taken? Or is it improbable that, in the present instance, it may have *been warded off* by the early exhibition of purgatives? These are questions which I confess myself not competent to solve. But this I know, that in *gastrodynia,* very acute spasmodic pain, shooting from the scrobiculus cordis to the spine, will almost infallibly yield to brisk purgatives; whereas opium will not only often *fail* to afford more than than the most momentary relief, but will sometimes *even bring it on*.

"My attention was forcibly drawn to this fact by two very striking cases, so long ago as the years 1793-4. It was not till after being completely baffled, so long as I trusted to what are called antispasmodics, and till after repeated observation of the relief afforded by purgatives, that my eyes were opened, so that I could see that every remission of the latter was followed by an exacerbation of the symptoms.

"One of these cases, more especially, I always look back to, as the great mark from which my subsequent views and practice took their direction; and later experience has so far confirmed the impression made by this first lesson, that if there be any point in medicine, on which, after having been engaged in Dispensary practice for near sixteen years, I have arrived at any certain conclusion, it is this, that in gastrodynia, and many other spasmodic affections, brisk purgatives will be found incomparably better *antispasmodics* than any of that tribe to which this epithet is usually applied. I believe, too, that their operation is *strictly* antispasmodic—that their *first* effect is to supersede the spasmodic action; for I have often known complete relief to be obtained *before* a stool was procured; insomuch, that I have more than once been asked by patients if I had not given them laudanum.

"Such having long been my general views, it may readily be conceived what pleasure I derived from the first perusal of Dr. Hamilton's valuable work; the steady light which he threw on the subject, enabled me to go on boldly in a path in which I had hitherto proceeded with timid steps, and as it were in the dark; and I am persuaded, that whoever will but give his practice a *full trial* in any of the cases for which he has recommended it, will be satisfied that he advances nothing for which he is not warranted by facts. His proposal for attempting the cure of tetanus by purgatives struck me immediately, as promising so much more favourably than any other plan of treatment I had before heard of, that I determined to give it a trial, if ever I should have an opportunity. But before any such opportunity occurred to myself, I saw it succeed completely in a case of trismus, under the direction of one of my colleagues; and that in the present instance, the cure must be ascribed to the purgatives, either wholly or in a great measure, seems incontrovertible, not only from the final result, but from the exacerbations consequent to every relaxation of the plan. I am now inclined to think, moreover, that the more drastic purges were laid aside for no sufficient reason, and that, had they been continued in moderate quantity, the patient's convalescence would have been more rapid. By the report of the 27th, he was himself of the same opinion; and indeed the effect of half of a powder and one dose of the mixture was, as it is stated, truly surprising; shewing, at the same time, how much the bowels had recovered of their natural susceptibility

 since since the time when it required so much stronger doses to produce the same effect. It seems farther evident, that the relief was not always in proportion to the quantity of even solid fæces evacuated. The more active purges appear literally to have possessed antispasmodic virtues more than the pills.

"The *quantity* of medicine taken, from first to last, is certainly very large, amounting, as far as can be ascertained, for the first twenty-five days, to calomel, gr. 320; scammony, gr. 340; gamboge, gr. 126; powder of jalap, ℥v. and ʒviiß; infusion of senna, with tincture, ℔x$\frac{2}{3}$; colocynth pill ℥iß, and gr. 45; of which the *greatest part was taken within the first week,* namely, calomel, 280; scammony, 260; gamboge, 110; jalap, ℥iii, gr. x. infusion, ℔5$\frac{5}{6}$. The quantity given in little more than forty-eight hours, from the morning of the 17th, is perhaps unprecedented in this country: amounting to—scammony, gr. 210; gamboge, 89; jalap, ℥i. and ℈iv.; infusion, ℔2$\frac{1}{3}$; calomel, gr. 80; —and all this without causing either sickness or griping, but on the contrary, with the most decided benefit.

"Some of my friends have found great difficulty in believing that the whole quantity was *fairly taken;* but I can assure them there is little or no room for doubt. Several of the *strongest* doses I gave to the patient myself; and very frequently, during the whole course of the disease, the medicine was taken in the presence of Mr. Bridge or me; and from the eager desire of the patient and his attendants to comply with all my directions, I have the fullest confidence in their reports of what took place in our absence. I was fearful indeed lest the distress he experienced in swallowing a dose of the mixture, on the evening of July 15th, should have deterred him from attempting it again; but his resolution was not to be shaken.

"And after all, it should be recollected, that it is not of *purgatives* alone that such extraordinary doses are required to produce any sensible effect in tetanus: where *opium* is the remedy relied on, the extent to which it is sometimes pushed, beyond the limits of a common dose, is equally or more enormous. Dr. Currie, in the case of Gardner, gave ℥viii. of the tincture in about fifty hours; of which not less than ℥v.ß were given in the last twenty-six hours!"

It should always be remembered, that the true lock jaw is a disease well marked in its whole progress; that the cause of death is not the contraction of the external muscles, but such an universality of the disease, that ultimately the muscles of those organs, whose actions are essential to life are affected; and a violent spasm of the heart terminates the scene. If the disease becomes chronic, that is, if it continues for a certain number of days without gradually extending to different muscles, the probability is, that the patient will recover. Whether the remedies used by Dr. Briggs prevented this extension, as he seems to suspect by his remarks on the ensiform cartilage, we pretend not to determine.

We

We are pleased, however, with the author's candour, in admitting, that he has no satisfactory proof of any wound, to which he could attribute the disease; and consequently the necessity of suspending any decision on these remedies, till trial shall be made on true *trismus traumaticus.*

Art. 5. *Observations on the different Methods recommended for Detecting minute Portions of Arsenic.* By John Bostock, M. D. Read before the Liverpool Medical Society.

The late events at Liverpool, probably, gave rise to this valuable paper; for valuable every paper must be from the pen of Dr. Bostock. The peculiar delicacy of his researches, and the accurate simplicity with which they are detailed, seem to point him out as the person who should instruct us in any similar question which may hereafter arise. It will be enough if we show the general result of the various experiments, and point out those instructions, which Dr. Bostock has left for those who may find it necessary to repeat them.

After a few general but well directed remarks, a description is given of the five methods proposed by different authors for detecting the presence of arsenic; namely,

1st. The precipitation of arsenic from any fluid in which it is dissolved by an alkaline hydro sulphuret.

2d. Its precipitation by sulphate of copper.

3d. The reduction of the oxyd into a metallic state, by heating it with extraneous substances in a glass tube.

4th. On the effect which arsenic has in whitening copper, when heated in contact with it.

And 5th. The peculiar odour which it exhales when evaporated from a heated surface. On each of these some general remarks follow, intended, as the author observes, to assist the scientific chemist, should he be suddenly called upon to state an opinion, without being in the habit of making frequent experiments.

" After a review of the different processes for detecting arsenic, it remains for us to determine on which of them the greatest confidence may be placed. I have no hesitation in giving it as my opinion, that the most convenient, the most delicate, and the most decisive process, is the one in which the green precipitate is formed by the addition of the sulphate of copper. The arsenic may be detected with certainty in a very minute quantity, as well in a fluid as in a solid state; no apparatus is requisite, nor is there any skill required on the part of the operator. When we are called upon, in a judicial investigation, to ascertain whether arsenic has been employed, we are generally obliged to perform our experiments on a small quantity of the substance which may be accidentally left in the cup or bottle, or to search for it among the contents of the stomach. In this latter case, particularly, it is only in minute quantity that we can expect to find it; and in order to separate it from the mass in which it is contained, we must have recourse to the solvent power of hot water.

 There

"There are two precautions which I should strongly recommend to every person who may be called upon to search for arsenic in a suspected substance. The first is, that in every experiment which is performed upon the unknown substance, a corresponding one should be made with ingredients of a known composition. If, for example, the test of the sulphate of copper be applied to a suspected fluid, the operator should at the same time add the potash and sulphate of copper to a portion of pure water, and afterwards he should repeat the process with the potash, sulphate of copper, and a given quantity of an arsenical solution. The effect of the re-agents will thus become familiar to him both with and without the arsenic, and he then will be enabled, with much more confidence, to state his opinion respecting the fluid under consideration. The second recommendation which I shall beg leave to offer, is, that the examination be never entered upon, until the substances that are intended to be employed as tests, and the necessary apparatus, are all in readiness, and all in a perfect state. In experiments of this kind, there should be *no allowances* made; and as it often happens that the quantity of the matter to be operated upon is small, none of it ought to be wasted upon imperfect trials."

ARTICLE 6.—*Case, in which the Operation of tying the Vena Saphena, for the Cure of an old Ulcer in the Leg, terminated fatally.* By HENRY OLDKNOW, Surgeon, Nottingham.

THIS is a most important paper, and we hope it will be the means of teaching many young practitioners, that however successfully they may have seen certain experimental operations performed, and certainly we must consider the present in no other light, yet that they are not to suppose no danger attends them, and consequently that they are to be undertaken without some urgency. That the operation was conducted by Mr. Oldknow with much accuracy, we cannot doubt, and we must admit that his subsequent treatment was highly judicious. If we have any objection, it is to performing it at all on a young subject of a plethoric habit. It must be admitted, that ulcers in the lower extremities, which were painful, and so frequently broke out afresh as to interfere with the necessary occupation of the patient, cannot be called a trifling disease. We admit too, that the operation has been proposed for cases much more trivial; and most of all we must express our gratitude for the candour of the author, in the readiness with which he communicates his ill success.

The operation was performed in the usual way, and seemed to be attended with no unfavourable symptom till the sixth day, when the patient "complained of a little pain on the inner side of the knee, in the course of the vein; but there was no external inflammation at that part; the lips of the wound began to separate. In the evening, he was suddenly seized with a violent rigour, succeeded by a hot fit, and symptoms of great vascular action, and some tendency to delirium. Pulse 130, hard and full; therefore sixteen sixteen ounces of blood were taken from the arm; it gave instantaneous relief, and was followed by the sweating stage, which continued several hours, and was succeeded by a state of quietude, soon, however, interrupted by a recurrence of the same train of violent symptoms, at first about once in twenty-four hours, but gradually increasing in frequency, and diminishing in strength, until nature became exhausted, death closing the scene twenty-two days from the operation. For the first four or five days after the rigor, the wound discharged pretty freely, and by making strong pressure about the knee, a little matter was forced from it. The inflammation crept gradually up the vein, which was evident from its peculiar cord-like feel, and from giving pain on pressure, until it reached the groin, the inferior part getting well as the superior became bad, so that the wound was nearly healed before death, the ligatures having separated about the fourteenth day. There was no tumefaction of the cellular membrane, no enlargement of the glands in the groin, no superficial inflammation on the thigh. There was, it is true, a slight redness of the skin when the poultice was removed, (for the thigh, along the course of the vein, was covered with cold poultice) which entirely vanished on exposure to the air. The medical treatment was strictly antiphlogistic; the patient was repeatedly bled, and with apparent relief every time, the blood being extremely sizy ever after the first rigor. Two days, however, previous to his death, the vital principle was so exhausted as to need the use of cordials.

"This is the result of an operation, which, I believe, is generally considered as a very trifling one, and not endangering the life of the patient; judge then my mortification at this unexpected termination. On inquiry, however, I find another fatal case has occurred in this neighbourhood, differing in its symptoms from the one I have related. Large collections of matter formed in the cellular membrane, along the course of the vein, as far as the groin, and the patient died two months after the operation, the fever assuming the form of an intermittent. In this case, the incision was made transversely, and the vein was tied by a single ligature, as mentioned by your correspondent. Many are the cases, where much inflammation has followed the operation, and no doubt many persons undergo the operation without any such occurrences; but even then, it is a question, whether it be attended with that permanent advantage we are led to expect from it. Ought we, therefore, in any such cases, to run the risk of the danger incurred by the operation? Mr. Home, in his book on ulcers, mentions symptoms of inflammation having taken place in a few instances; but he is more inclined to attribute them to the unhealthiness of large hospitals, than to any immediate effect of the operation; and in my opinion, his book is calculated to impress the minds of medical men, with too little importance in regard to the operation, and its consequences. In the above case, you will perceive the inflammation was confined to the vein itself. Are we then, with Mr.

 Hunter,

Hunter, to attribute the death of the patient to the inflammation extending to the cavities of the heart, or to an inflammatory exhalation from the inner surface of the vein, which being carried into the circulation, acts a constant source of irritation to the heart and brain, and thus produces the disease? I think, at least, such an opinion is not irrational, knowing as we do the deleterious effects which all the animal secretions, when injected into the veins, have upon the system."

Article 7.—*Case of Flooding, between the Seventh and Eighth Month of Pregnancy, occasioned by the Attachment of the Placenta to the Mouth of the Womb, where the Woman was obliged to be delivered without Natural Pains, and both Mother and Child recovered.* By William Robertson, Surgeon, Kelso.

In this instance a part of the placenta had been protruded through the os uteri, so long that it was become putrid, before Mr. Robertson attempted the premature delivery. There could therefore be little hopes of escape for the woman, but by such an attempt. No efforts for this purpose being made by Nature, Mr. Robertson succeeded in bringing the child by the feet, and thus preserved the lives of both.

Article 8.—*Case of Aneurism of the Femoral Artery.* By David Hossack, m. d. Professor of Materia Medica and Botany, in Columbia College, &c.

In this case the artery was tied by two ligatures, the highest about two inches from the groin. An assistant compressed the artery, as there was not sufficient space for the tourniquet. The ligatures were distant about an inch, and the vessel afterwards divided near the lowest ligature. The parts healed without ulceration, and the patient recovered.

Pains were taken to prevent too great a separation of the artery from the surrounding parts, that the vessel might be supported till its cavity should be obliterated: a caution which seems reasonable, as it is well known that the vasa vasorum arise not from the arteries themselves, but from the surrounding parts. However it should be recollected, that the retractile power of the arteries, and the inherent power of sudden inosculation, and even the quick formation of small vessels, are probably sufficient to feed the arteries, especially as we find them considerably drawn out after amputation without injury. It is probable, when sloughing or ulceration takes place after the tying of an artery, that it should be ascribed to some other cause. On this important, and by no means sufficiently understood subject, we recommend the perusal of Mr. Jones's valuable experiments.

Article 9.—*Observations on Anchylosis.* By Richard Carmichael, Esq. Surgeon, Dublin.

The object of this paper is to recommend, in all cases of suppurated joint, that an attempt should be made to heal by anchylosis rather than hastily remove the part. Instances are given of the author's success in such attempts

Article

ARTICLE 10.—*Medical Reports for Nottingham, from March* 1807 *to March* 1808. By JAMES CLARKE, M. D. Physician to the General Hospital, and to the Vaccine Institution.

THIS report commences with a very reasonable complaint against the expensive manner in which medical books are published. Probably this, like many other evils, will find its cure, or the price of books, like all other articles, will find its level. However, it must be admitted, that the medical man, who industriously gives the result of a laborious enquiry pursued for a series of years, is entitled to some return, and the public are not generous enough to pay according to intrinsic value, but require a certain portion of paper, and even sometimes an engraving for their money. This might be illustrated by one branch of the medical profession, who, from custom, not being paid for their visits or advice, are under the necessity of seeking for their fair returns by methods less agreeable to themselves.

Dr. Clarke conceives, that the public has a strong claim on dissectors, to communicate all their knowledge. "Such men," says he, "have a privilege which is granted to them alone." If by this is meant the authorized *Professors* of Anatomy, there may be some justice in the remark; if it means only our London teachers, we know of no privileges they can boast which are not open to all. Their schools are either raised by themselves or their predecessors, by means which others may pursue, and are daily pursuing.

The present paper contains a series of remarks on the table contained in a former number; we shall notice only those which seem entitled to the reader's attention. A case of gout in a young subject was treated successfully with venæsection, immersion of the part in cold water, and an aperient remedy.

Two cases follow with the dissection, which are related with sufficient minuteness during the time they remained in the hospital, but do not furnish any particularly useful practical lessons. We wish the nosological part had been omitted. We know of no use that the term hæpatalgia *scirrhosa* can serve. An interesting case follows of a sac of hydatids attached to the liver, but the relation of the dissection is somewhat obscure. Some valuable remarks are offered on consumption; and in this benevolent age, in which so much notice is taken of our sable brethren, we cannot help wishing that the whites might come in for at least some share of the same attention.

"*Phthisis* makes great ravages in this manufacturing district. The close confinement in small unhealthy apartments, irregularly heated, and scarcely ventilated, must make an unfavourable impression on the constitution. In this town consumption preys chiefly on the labouring part of the community. Their children from the age of five are placed at the tambour frame, or employed to seam or cheven stockings, occupations in which they must necessarily remain many hours in a bending posture; and too often their only relaxation is an hour or two in the evening, exposed to the damp air, air, and a few short hours devoted to sleep. At this work the boys continue to the age of eight or sometimes twelve, when they are put in the stocking-frame. This obliges the body to assume a very different position; the arms are now thrown outwards, and the chest (if not contracted past remedy) forcibly expanded. Many continue several years unable to perform any heavy work, from weakness of the chest and arms; but they now enjoy a little more liberty, should they fortunately meet with a humane master. They are still, however, when at work, penned up in close rooms, warmed perhaps by a stove, and badly ventilated; they in consequence become more susceptible of cold, often leaving the work-room covered with perspiration; and the effects are in proportion more serious, as the nature of their employment, if it does not dispose to, tends not a little to increase any incipient inflammation of the chest.

"The females ever continue in the same prone posture; are generally high shouldered, and by their figure are easily distinguished from females whose employments are of a different nature. The rooms in which they work are much less objectionable than those for the men; but their bed-chambers are generally very indifferent, and six or more sleep in one small garret, with the air carefully excluded;

> "Save one dull pane, that, coarsely patch'd, gives way
> "To the rude tempest, yet excludes the day."*

"But what is equally destructive to the health of this community, is the carelessness with which they inhabit newly built houses, in which they often live and sleep whilst the plaster is quite wet on the walls, and indeed sometimes before the windows have been placed in the frames. These causes would be sufficient to account for the prevalence of consumption in this town.

"Some in the incipient state of the disease, when admitted into the hospital, have been restored with very little medical assistance; a recovery which with justice may be attributed to the regular and genial warmth of the wards, and the respiring of pure warm air; the lungs being preserved from moist air, or the still more deleterious effects arising from open coal fires; to which foreigners attribute the great prevalence of consumption in this country."

---

### *Trial of Mr. Angus, and subsequent Pamphlets.*

( Concluded from our last. )

At the conclusion of this article in our last Number we remarked, that though both parties quote Mr. Hunter's words correctly from the same paper, and apparently on the same subject, yet they are in direct contradiction to each other. Mr. Carson remarks, that

* Crabbe's Poems, p. 12.

that Mr. Hunter has described the stomach, when digested by its own secretion, almost in the words used by these Gentlemen in their Report on Miss Burns's stomach, viz. "The edges," says Mr. Hunter, "round this opening appear to be half dissolved, very much like that kind of dissolution which fleshy parts undergo when half digested in the stomach, or when dissolved by a caustic alkali; viz. pulpy, tender, and ragged."

"The natural structure of the coats of the stomach for a considerable space round this opening," says the Report, "was destroyed, and they were so soft, pulpy, and tender, that they tore with the slightest touch."

Here Dr. Carson has certainly the advantage; but let us see what the Reporters say on this subject.

In the fifth objection made by the above gentlemen, it is said, "because the appearances in the stomach did not correspond with the effects of the gastric juice upon the stomach, as described by Mr. J. H. the only writer who has described them. His description is as follows: By comparing the inner surface of the great end of the stomach, with any other part of the inner surface, the difference will be obvious. "The sound part appears soft, spongy, and granulated, *without distinct blood vessels*, opake and thick; the other part" (that is, the part acted upon by the gastric juice) "appears smooth, thin, and more transparent, the vessels are seen ramifying on the surface, and upon squeezing the blood which they contain, from the larger branches to the smallest, it will pass out at the digested ends of the vessels, and appear like drops upon the inner surface." Dr. Carson in his remarks on this passage, says,

"The fifth objection is, that the appearances in Miss Burns's stomach did not correspond with the effects of the gastric juice upon the stomach, as described by Mr. John Hunter. This objection certainly surprises me not a little. The appearances described by Mr. Hunter, were, almost word for word, the same with those described by the gentlemen in their evidence at Lancaster, in this case. The edges of the hole, they say, were pulpy, tender, ragged, and broken down. The edges of the holes in Mr. Hunter's cases were pulpy, tender, and ragged. The parts about the hole had the appearance of being acted upon by the caustic alkali; and as I mentioned in evidence, the parts about the aperture in this stomach were described to me by Dr. Gerard, as having the appearance of being acted upon by the caustic alkali. The only difference seems to be, that the blood could be squeezed out of the ends of the vessels in Mr. Hunter's cases, but not in this. But this difference is purely accidental, and arises from the following cause: The part of the stomach which Mr. Hunter observed to be perforated, was in the large curvature opposite to the spleen. Now, it is well known to all anatomists, that the vessels called vasa brevia pass from the spleen to the stomach and spread on its surface at this part. The blood-vessels at this part are large large and numerous. When the stomach is full, as was the case in these instances, these vessels are known to be more distended with blood than when the stomach is empty. This accounts for the quantity of blood that could be squeezed out of the divided vessels in the cases observed by Mr. Hunter. But in this instance the perforation was much nearer the pylorus on the same curvature where it is known the blood-vessels of the stomach are very small. Hence little or no blood could be squeezed out of the ends of the vessels."

The assertion, that the perforation was nearer the pylorus is equally gratuitous and unnecessary. If the edges of the hole were dissolved, as above described, neither blood nor blood-vessels could be discovered in a part dissolved by caustic alkali, so as to become "pulpy, tender, and ragged."

Whoever reads Mr. Hunter with the care taken by that gentleman to explain himself, will see, that he is describing two distinct effects in the same part of different stomachs. In those, where its digestion has proceeded so far as to perforate the stomach, the edges of the perforation are, as above mentioned, pulpy, &c. These cases are certainly not frequent, and in all those described by Mr. Hunter, the patients died by a violent or sudden death. But another appearance he speaks of as so *very common*, that he tell us, "there are very few dead bodies in which the stomach is not, at its great end, in some degree digested; and one who is acquainted with dissections, can easily trace the gradations from the smallest [degree of digestion] to the greatest." Then follow the words as quoted in the Report.

By this it is evident, the appearance of blood-vessels from the extremities of which blood may be squeezed, must relate to the stomach in all but "a very few subjects," though the appearance may be overlooked by such as are not so well acquainted with dissections as that illustrious physiologist. In these the patient having died, without being under those circumstances which are necessary to produce a complete digestion of the stomach,* a part only has been eroded on the surface, and this erosion has been sufficient to destroy either the whole diameter or the upper surface of small blood-vessels; in consequence of which, the blood pressed "from the larger branches to the smaller, will be found to pass out at the digested ends of the vessels, and appear like drops on the inner surface."

We shall take no notice of Dr. Carson's mode of accounting for this dissolution of the stomach, by Sir J. Pringle's fermentation; not, because it differs from Mr. Hunter's explanation, which as he seems to suggest, is a sufficient objection with some people, but, because

* For an account of the circumstances under which the stomach of a rabbit may be made to digest itself, see our Journal, vol. xvii. p. 569, and the work there referred to.

because it appears to us at war with common sense and daily experience. It is, however, worth remarking, that Sir John was himself so sensible of the justness of Mr. Hunter's opinion, and so little disposed to impute the appearance in the stomach they examined together to any other cause, that he would not suffer Mr. H. to delay his relation of the fact with his remarks "to a future period, but desired they might be given by themselves, as it would be of use in the examination of dead bodies."

On the other hand, we are not satisfied with the Report of these gentlemen. There are indeed, two ways in which the patient might have been destroyed by poison, and the stomach have appeared as described. Had a substance been swallowed sufficiently stimulating to excite high inflammation, afterwards terminating in mortification, the part thus affected being dead, might have been digested whilst the rest of the stomach might retain its natural appearance, for after mortificaiion the inflammation would cease, nor can be ascertained how far it might have extended.

If a substance also proved poisonous in the manner described in the experiments on the dogs, the state of the patient immediately, and indeed for some hours before death, was favourable for such a solution of the stomach. Miss B. had so far recovered as to keep on her stomach what she swallowed, and expressed a choice in what she took. By this it is probable, that the secretion of the stomach returned; and if, under these circumstances, she died, nothing could be more reasonable than that the secretion still remaining in the very substance of the stomach, or applied to its immediate surface, should have digested that organ as soon as it was dead. It is no objection to say, that in the short space between the time that the violent symptoms ceased, and that death took place, it is improbable any healthy action could commence; because we know, that in many instances, an apparent cessation of every dangerous symptom is the forerunner of death. Some of our readers will, perhaps, be able to turn to the case of a Dutch nobleman under the care of Boerhaave, but related by Zimmerman, or some other continental physician. In this instance, the patient was seized with violent pains in the gastric region immediately after swallowing a particularly hearty dinner. Every means of relief failed, till suddenly all the symptoms ceased, and the patient felt the return of hæmorrhoids, to which he had been subject. So well was he satisfied with his situation, that he began to reproach his physician for not having thought of this mode of relief, that is, for not endeavouring to relieve him by inducing the piles, to the spontaneous return of which he imputed his cure. A few hours, however, convinced his friends of the mistake, when they beheld him a corpse. On opening the body, part of his dinner was found loose in the abdomen, and death was imputed to the bursting of the stomach from over distention.

But whilst candour induces us thus to reconcile ourselves to the language of the Report, and the subsequent reasonings of the Reporters, porters, we know not how to express our feelings at the conduct of Dr. Carson. Every one knows, that in describing events and appearances, to those whose habits and inquiries have not been directed to physical subjects, it is absolutely necessary, sometimes, to use language different from what may be deemed strictly technical: That is, to mention results from causes without tracing all the intermediate processes. Under that impression, the gentlemen seem to have drawn up their Report relative to the effect of the acrid poison to which they imputed the death of the deceased. Had they been aware that their Report was to be scrutinized by a professional man, there cannot be a doubt that they would have modelled it in such a manner, as to have met every objection, though probably not every cavil. Their astonishment therefore, at hearing Mr. Carson's objections to their Report, stated for the first time before a judge and jury, may easily be imagined. After a preparation of several weeks, we find him before the court, contradicting gentlemen on a subject, on which, as they conceived, there was only one opinion in Liverpool, they had never prepared themselves to meet any objection. All this is perfectly accordant with forensic proceedings, the pleaders in which, profess only to aim at persuasion for the moment, regardless of every thing but success. But medicine is an inquiry after truth. It may, perhaps, be urged, that in the present instance, the wish of Dr. Carson extended no further than to rescue the prisoner, as a lawyer would his client. We shall not take upon ourselves to determine how far this may be justifiable. But there must be a difference between a pleading and a deposition. Still it may be said, that all Dr. Carson urged was within the scale of possibility; and when the life of a fellow creature is at stake, it may be right to make the best of every favourable argument. In that case, we wish Dr. Carson had stopped with his deposition before the court, and not promised a publication, nor published his Remarks.

We shall conclude this discussion on the digestion of the stomach, with offering a simple conjecture of our own. From the most minute examination of the whole question, we are ready to admit that the proof of poison is deficient; that the violent vomiting might have been occasioned by a sympathetic affection of the stomach with the uterus. Whether the latter organ was unnaturally stimulated we pretend not to determine, but we cannot help expressing our surprise, that any one doubt could have been entertained concerning the pregnancy and recent delivery of the deceased. However, supposing so very remarkable a coincidence as a death immediately after the expulsion of hydatids, and before the womb had contracted, an appearance, similar to that which is seen soon after the separation of the placenta, and, that the same subject should exhibit a virgin ovarium with a corpus luteum; in short, that the appearaces should be so striking, as to induce an unanimous decision from the best informed men in London, that such a subject a subject must have been pregnant and recently delivered. Supposing all this to have been the effect of hydatids, still a difficulty remains. What became of these hydatids after their expulsion? Why were they concealed? Who took them away? It may be said that this is not a medical question. To this we answer, that under under such extraordinary events, we are obliged to call in every means of information. And, if it is really true, that neither fœtus nor hydatids were expelled, we are obliged to look for some more remote cause, for appearances which, under common circumstances, would have been sufficient to have satisfied any one of a real and regular pregnancy, as well as of a delivery.

The last pamphlet of this Controversy is, "Mr. Dawson's Exposure of the false Statement," contained in Dr. Carson's. We forbore to offer any personal extract from the last, for the reasons mentioned above; it may therefore, seem unfair to offer any from the present, which are chiefly in answer to them. Indeed, such could only relate to personalities, which would be uninteresting every where, excepting in the meridian of Liverpool, where the whole is probably too well known.

We must however in general remark, that we felt great interest in the perusal of Mr. Dawson's letter. The anatomical remarks are highly creditable to the author, and the indignation expressed at some unjust surmises of his antagonist, seems to flow from a mind conscious of its own purity, and little acquainted with controversial animosities. Mr. Dawson takes no anatomical notice but of the appearances of the uterus and its appendages.

Thus have we offered as succint an account of this business, as our limits and impartiality would admit. We have received several communications on the subject, some of which are anonymous and too personal to be admitted; others we have deferred till we could render them interesting or intelligible to the common reader.

